# Transactions of American Medical Association, Vol. XXXI. 1880

**Published:** 1881-02

**Authors:** 


*The Transactions of the American Medical Association*, Volume XXXI, 1880. Containing the minutes of the Thirty-first Annual Meeting, held in New York City, June 1st, 2nd, 3d and 4th, 1880.

This Report of Proceedings contains many valuable papers on interesting subjects, and embraces eleven hundred and fifty-three pages.

